# High-risk and multiple human papillomavirus (HPV) infections in cancer-free Jamaican women

**DOI:** 10.1186/1750-9378-4-S1-S11

**Published:** 2009-02-10

**Authors:** Angela Watt, David Garwood, Maria Jackson, Novie Younger, Camille Ragin, Monica Smikle, Horace Fletcher, Norma McFarlane-Anderson

**Affiliations:** 1 Department of Basic Medical Sciences, Faculty of Medical Sciences, University of the West Indies, Kingston, Jamaica; 2 Department of Community Health & Psychiatry, University of the West Indies, Kingston, Jamaica; 3 Epidemiology Research Unit, Tropical Medicine Research Institute, University of the West Indies, Kingston, Jamaica; 4 Department of Epidemiology and Division of Cancer Prevention and Population Science, University of Pittsburgh, Pittsburgh, PA 15232, USA; 5 Department of Microbiology, University of the West Indies, Kingston, Jamaica; 6 Department of Obstetrics and Gynaecology, University of the West Indies, Kingston, Jamaica; 7 Department of Epidemiology and Biostatistics, Downstate School of Public Health, State University of New York, USA

## Abstract

**Background:**

Vaccines, that target human papillomavirus (HPV) high risk genotypes 16 and 18, have recently been developed. This study was aimed at determining genotypes commonly found in high-risk and multiple-HPV infections in Jamaican women. Two hundred and fifty three (253) women were enrolled in the study. Of these, 120 pregnant women, aged 15–44 years, were recruited from the Ante Natal Clinic at the University Hospital of the West Indies and 116 non-pregnant, aged 19–83, from a family practice in Western Jamaica. Cervical cell samples were collected from the women and HPV DNA was detected using Polymerase Chain Reaction and Reverse Line Hybridization. HPV genotypes were assessed in 236 women. Data were collected from January 2003 to October 2006.

**Results:**

HPV DNA was detected in 87.7% (207/236) and of these 80.2% were positive for high-risk types. The most common high-risk HPV types were: HPV 45 (21.7%), HPV 58 (18.8%), HPV 16 (18.4%), HPV 35 (15.0%), HPV 18 (14.5%), HPV 52 (12.0%) and HPV 51(11.1%). Other high-risk types were present in frequencies of 1.4% – 7.2%.

Multivariate regression analyses showed that bacterial vaginosis predicted the presence of multiple infections (OR 3.51; CI, 1.26–9.82) and that alcohol use (OR 0.31; CI, 0.15–0.85) and age at first sexual encounter (12–15 years: OR 3.56; CI, 1.41–9.12; 16–19 years, OR 3.53, CI, 1.22–10.23) were significantly associated with high risk infections. Cervical cytology was normal in the majority of women despite the presence of high-risk and multiple infections.

**Conclusion:**

HPV genotype distribution in this group of Jamaican women differs from the patterns found in Europe, North America and some parts of Asia. It may be necessary therefore to consider development of other vaccines which target genotypes found in our and similar populations. HPV genotyping as well as Pap smears should be considered.

## Background

Infection with oncogenic human papillomavirus (HPV) types is the primary cause of cervical cancer, the second most common cancer among women worldwide [1]. In developing countries, cervical cancer is second to breast cancer as the cause of cancer deaths in women of reproductive age [2] and is still a significant public health problem among women in the Caribbean [3]. To date, over 200 HPV types of which 40 types infect the genital tract, have been identified worldwide [4]. The most recent data show that in Jamaica, the age-specific incidence is 19/100,000 [5]. HPV genotypes 16, 18 and 45 were identified in Colposcopy clinic patients [6]. The development of a prophylactic vaccine that targets HPV 6, 11, 16 and 18 has increased interest in the ethnic and geographical distributions of HPV genotypes. This study focuses on the presence of high-risk and multiple HPV infections in a population of cancer-free Jamaican women and seeks to determine whether HPV infection is associated with lifestyle and sexual practices.

## Materials and methods

### Study population

Two hundred and thirty six women were enrolled in the study: 120 pregnant women aged 15 – 44 years (median age 27.5 years) at their first antenatal clinic visit to the University Hospital of the West Indies (UHWI) in Kingston, Jamaica and 116 non-pregnant women (median age 35 years) attending a family practice in Western Jamaica. Selection of the participants was consecutive. Participants were asked to complete a questionnaire that assessed information on socio-demographic factors, sexual practices and history of sexually transmitted infections (STI). Ethical approval was obtained from the University of the West Indies (UWI)/UHWI Ethical Committee. Informed consent was obtained from all participants. Participants were recruited January 2003 – October 2006.

### Sample collection

At enrollment, a routine gynecological examination was conducted and cervical specimens were collected first for Papanicolaou (Pap) smear and for HPV testing. The cervical cells for HPV testing were taken from the endocervix with one to two cytobrushes. The brushes were then agitated in a 15 ml tube containing 1 ml of specimen transport medium (Roche Diagnostics, Germany) for several seconds to release the cells. The cervical cells were stored at -20°C until further processing. Cytology results were obtained from Pap smears. Cytological abnormalities were classified according to the Bethesda classification system as within normal limits or reactive cellular changes (normal); atypical squamous cells of undetermined significance (ASCUS); cervical intraepithelial neoplasia I (CINI); cervical intraepithelial neoplasia II (CINII); cervical intraepithelial neoplasia III (CINIII) and carcinoma.

#### Detection and genotyping of HPV

Briefly, 250 μl aliquot of the cervical cell sample collected in the specimen transport medium was lysed in the presence of proteinase K. Isolation and purification of the released nucleic acid were carried out using columns and elution reagents and the presence of HPV DNA was detected using PCR-based methods: AMPLICOR [7] and the Roche Linear Array HPV genotyping test protocol [8]. This method uses biotinylated primers to define the specific L1 region of the HPV genome and is able to detect 37 HPV genotypes (6, 11, 16, 18, 26, 31, 33, 35, 39, 40, 42, 45, 51, 52, 53, 54, 55, 56, 58, 59, 61, 62, 64, 66, 67, 68, 69, 70, 71, 72, 73, 81, 82, 83, 84, IS39 and CP6108). The HPV genotype strips contain probe lines specific to the detection of the 37 HPV types and 2 concentrations of the β-globin control probe. For detection, the PCR products were denatured and then 75 μl of the denatured amplicon was added to the probe strip in a hybridization buffer. After the final wash, the strips were incubated in a buffer containing streptavidin-horseradish peroxidase conjugate to facilitate detection of the different HPV types. The buffer was then removed by vacuum aspiration, and the strips rinsed in a citrate solution. Colour development was activated by incubation in a mixture of hydrogen peroxide in citrate solution and tetramethylbenzidine in dimethylformamide for 5 minutes, on a rotating platform at 60 rpm. Strip interpretation was performed using a labeled reference guide, with lines indicating the position of each probe relative to a HPV type reference mark. We considered HPV genotypes 16, 18, 31, 33, 35, 39, 45, 51, 52, 56, 58, 59, and 68 as primary carcinogenic (high-risk) types and all others as non-oncogenic (low-risk) types [9]. Ten subjects who were HPV-positive (using the AMPLICOR method) were not assigned to a genotype.

### Statistical analysis

Chi-square statistics were used in the cross-classification of the presence of HPV infection and lifestyle/sexual practices characteristics. Multiple logistic regression analyses were used to examine the association of sexual and lifestyle behaviours with HPV infections. Statistical analyses were performed by the Statistical Package for Social Sciences (SPSS version 12).

## Results

Table 1 displays the distribution of HPV infections according to demographic, lifestyle and sexual practice variables. Substantially more pregnant than non-pregnant women had HR-HPV infections and few women who reported that they had never been pregnant had HR-HPV infections. Almost all the HPV-positive women were diagnosed with normal cytology. Alcohol consumption was lowest in high-risk HPV-positive women. HPV prevalence did not vary significantly by age, number of sexual partners, age at first sexual encounter, hormonal contraceptive use, parity or reported history of sexually transmitted diseases.

**Table 1:** Comparison of demographic, lifestyle and sexual practice variables in HPV-negative and HPV-positive women.

	**HPV-negative** **n = 29**	**Low risk** **HPV-positive** **n = 39**	**High-risk HPV-positive** **n = 158**
Pregnancy:*			
Pregnant	44.8 (13)	51.3 (20)	65.8 (104)
Non-pregnant	55.2 (16)	48.7 (19)	3402 (54)
Gravidity:			
0	13.8 (4)	10.3 (4)	5.7 (9)
1–2	48.3 (14)	43.6 (17)	50.0 (79)
3 or more	37.9 (11)	46.3 (18)	44.3 (70)
Parity:			
0	34.5 (10)	35.9 (14)	34.0 (52)
1–2	34.5 (10)	46.2 (18)	51.0 (78)
3 or more	31.0 (9)	17.9 (7)	15.0 (23)
Age at first sexual intercourse:†			
<16 years	36.0 (9)	16.7 (6)	25.2 (35)
16 – 19	18.0 (12)	61.1 (22)	66.9 993)
20 years older:	16.0 (4)	22.2 (8)	7.9 (11)
Cytology diagnosis:			
Normal	91.3 (21)	96.9 (31)	93.6 (131)
Dysplasia	8.7 (2)	3.1 (1)	6.4 (9)
Bacterial vaginosis	13.0 (3)	3.1 (1)	14.3 (20)
Reported sexually transmitted infections			
Yes	86.2 (25)	76.9 (30)	79.2 (122)
No	13.8 (4)	23.1 (90	20.8 (32)
Age groups:			
<25 years	27.6 (8)	28.6 (10)	28.8 (44)
25 – 34	41.4 (12)	42.9 (15)	41.2 (63)
35 – 44	20.7 (6)	14.3 (5)	24.8 (38)
45 or older	10.3 (3)	14.3 (5)	5.2 (8)
Smoker			
Yes	3.4 (1)	5.1 (2)	2.5 (4)
No	96.6 (28)	94.9 (37)	97.5 (153)
Alcohol consumption:*			
Yes	31.0 (9)	28.2 (11)	13.5 (21)
No	69.0 (20)	71.8 (28)	86.5 (134)
Hormonal contraceptive use			
Yes	48.3 (14)	67.6 (25)	57.6 (87)
No	51.7 (15)	32.4 (12)	42.4 (64)
Number of sexual partners:			
1 – 5	72.4 (21)	94.9 (37)	82.1 (128)
6 – 15	20.7 (6)	5.1 (2)	14.7 (23)
>16	609 (2)	0	3.2 (5)

† Subjects missing

Normal – no abnormal changes.

Dysplasia – abnormal cervical changes including ASCUS, CIN I-III;

ASCUS – atypical squamous cells of undetermined significance

CIN – cervical intraepithelial neoplasia

*X^2^; P < 0.05

The median age of the pregnant and non-pregnant women was 27.5 years and 35 years respectively. Approximately two-thirds of women reported having attained secondary education and less than 10% were employed in professional occupations. HPV DNA was detected in 87.7% of the sample; the virus was identified in 90.5% of pregnant and 83.8% of non-pregnant women.

Cytology results were obtained for 120 pregnant women and 92 non-pregnant women. Women with cervical dysplasia (14/207) had no consistent specific HPV types present, but many had multiple infections consisting of high- and low-risk HPV types, of which HPV types 16 and 58 were most common (data not shown). The majority of women with high-risk infection (93.6%) were diagnosed with normal cytology (data not shown).

**Figure 1:**
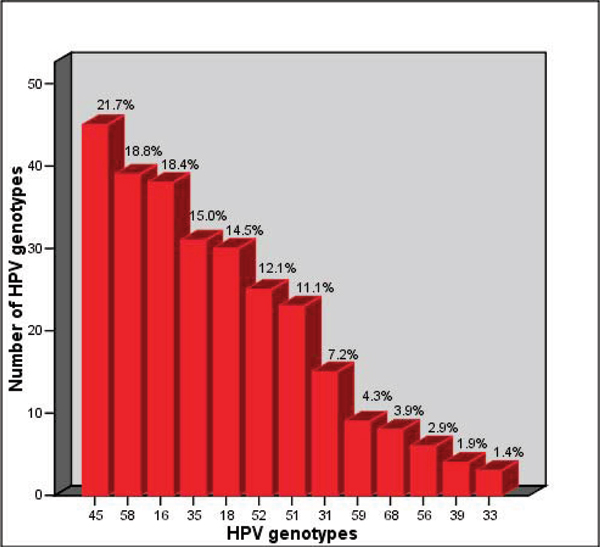
Frequencies of the most common high-risk HPV genotypes in pregnant and non-pregnant women.

Eighty percent (80.2%) of HPV-positive women had HR-HPV infections and of these 60.9% had multiple infections. The percentages of low-risk and single infections were 61.8% and 34.3% respectively. Figure 1 shows the most common HR-HPV genotypes found in HPV-positive women. HPV 45 (21.7%) occurred more frequently than other HR-types, followed by HPV 58, 16, 35 and 18 at 18.8%, 18.4%, 15.0% and 14.5%, respectively. Other HR-HPV genotypes were found in frequencies of 1.4% – 7.2%.

There was a wide distribution of genotypes in both groups of women (36 genotypes detected), although certain genotypes occurred more frequently in pregnant women. High-risk types 16, 18, 51, 52, 58, 59, and 68 were found in greater frequencies in pregnant women than non-pregnant women. Pregnant women also had the highest frequency of HR-HPV DNA (83.8%) and multiple infections (66.9%) compared to non-pregnant women (HR-HPV DNA, 77.1%; multiple infections, 51.8%). Twice as many pregnant as non-pregnant women had multiple genotypes present (Table 2).

**Table 2:** Number of HPV genotypes present in multiple infections in pregnant and non-pregnant HPV-positive women

	**HPV-positive women**	
	Non-pregnant women (n = 43)	Pregnant women (n = 83)
**Number of genotypes**: n (%)		
Two genotypes	16 (37.2)	29 (35.0)
Three genotypes	10 (23.3)	20 (24.1)
>3 genotypes	17 (39.5)	34 (40.9)

Examination of the presence of multiple infections by the above-mentioned variables showed that women who were pregnant (pregnant, 65.9%) or less than 34 years old (less than 25 years, 34.2%; 25 – 34 years, 42.5%; 35–44 years, 17.5%; 45 years or older, 5.8%) or were diagnosed with bacterial vaginosis (17.1%) were more likely to be infected by multiple genotypes. The patterns of multiple infections by all other variables were similar to those with high-risk HPV-positive infections (data not shown).

Univariate regression analyses were used to examine associations of behavioural and reproductive factors with three outcomes: HPV positivity, the presence of multiple infections and high-risk HPV infections. Women who consumed alcohol (OR, 0.39; CI, 0.20–0.77) or who were 20 years or older at first sexual intercourse (OR, 0.37; CI, 0.14–1.00) were at decreased risk of high-risk infections. The presence of bacterial vaginosis at screening (OR, 3.51; CI, 1.26 – 9.82), being 25 years or younger (OR, 3.70; CI, 1.04–9.07) and pregnancy (OR, 1.72; CI, 1.00–3.04) increased the risk of multiple infections. There were no associations of any behavioural or reproductive variables with HPV positivity.

In multivariate logistic regression analyses, controlling for age as a potential confounder, we examined significantly associated variables with high-risk HPV infections and multiple infections (Table 3). Two variables were associated with high-risk HPV infections: women who consumed alcohol showed decreased risk (OR, 0.31; CI, 0.15–0.85) whereas being younger at first sexual encounter was associated with increased risk (12–15 years: OR, 3.56; CI, 1.41–9.12; 16 – 19 years, OR 3.54, CI, 1.22–10.23) (data not shown). Regarding multiple infections, being pregnant lost its significance and only bacterial vaginosis predicted the presence of several genotypes.

**Table 3:** Odds ratios (Confidence Interval) for associations with high-risk HPV infections^a ^and multiple infections^b ^in Jamaican women.

	**Odds ratios (CI)**
**High-risk HPV infections:**	
Alcohol intake	
No	1.0 (reference)
Yes	0.31 (0.15–0.85)
Age at first sexual encounter	
20 years or older	1.0 (reference)
16 – 19	3.56 (1.41–9.12)
12 – 15	3.54 (1.22–10.23)
	
**Multiple infections:**	
Pregnancy status	
Non-pregnant	1.0 (reference)
Pregnant	1.67 (0.92, 3.03)
Bacterial vaginosis	
No	1.0 (reference)
Yes	2.76 (1.01, 8.02)

^a ^Adjusted for age of participant, age at first sexual intercourse, smoking (yes/no), alcohol use (yes/no), bacterial vaginosis (yes/no), and pregnancy status (non-pregnant/pregnant).

^b ^Adjusted for age.

## Discussion

This study which used cervical cells, a good medium for detection of HPV DNA [10 ] and the Gravitt et al. detection method [8], the most sensitive method to date, is unique in its evaluation of high-risk and multiple HPV infections in cancer-free Jamaican women. This investigation revealed high rates of infections, high levels of multiple infections that included both high- and low-risk genotypes and a pattern of infection dissimilar to those described in predominantly European populations.

The most important finding was that unlike the genotype distribution patterns seen in North America, Europe and some parts of Asia [11,12] HPV types 16 and 18 were not the most common high-risk genotypes. In our population, HPV types 45 and 58 accounted for 40.5% of the genotypes. Other groups, e.g. Trinidad and Tobago, Cuba and parts of Africa [13-18], have also reported different distributions of genotypes indicating that types 16 and 18 were not predominant in these populations. The recently developed prophylactic vaccines may therefore not be efficacious in our and similar populations.

Despite the predominance of genotypes other than types 45 and 58 in our population, it is not known whether these genotypes are important for the development of cancer. Several authors have suggested that HPV types 51, 52, 56 and 58 may act with HPV 16 in the development of dysplasia [19-21]. Multiple infections with HPV -16 and -58 were detected in 50% (7/14) of women with cervical dysplasia and earlier reports demonstrated the presence of types 16 and 45 in Colposcopy patients in Jamaica [6]. Further investigations are needed to determine the types that persist and are responsible for dysplasia and its sequelae in our population.

The majority of the women (93.2%) including those with high-risk infections had normal cytology. It has been suggested that cytological abnormalities may resolve before clearance of high-risk genotypes [22], and this could explain why in this study the majority of women positive for high-risk HPV genotypes had no evidence of dysplasia. Our findings were consistent with the International Agency for Research on Cancer (IARC) reports that the most common HPV types in women without cervical abnormalities were HPV-16, -42, -58, -31, -18, -56, -81, -35, -33, -45 and -52 [23]. The fact that women with normal Pap smears may be positive for high-risk HPV genotypes, suggests that in addition to Pap smears, HPV testing should be considered.

Various studies have reported prevalence ranging from 14% – 90% [12]. Our rates of infection (87.7%) are similar to those reported in USA inner city adolescents (90%) [24] and in HIV-positive Brazilian [25] and Zambian [18] women (only 2 women were diagnosed HIV-positive in this study group), but are higher than those reported from several West African countries (Gabon, 46%; Tanzania, 34% and Gambia, 13%) [15-17] as well as other countries in the Caribbean (Tobago, 35.4% and Cuba 21.7%) [13,14]. These findings would argue against a role for genetic predisposition since these groups share some common ancestry. Differences in prevalence between this study and an earlier investigation in Jamaica (1995) which showed a 28.7% HPV infection prevalence among women attending a Sexually Transmitted Disease Clinic [26] may be explained in part by differences in methodologies.

In our sample younger women were more likely to be positive with high risk infections or to have multiple infections. However, age, was not a significant predictor of any of the outcome variables. This is in contrast to studies that show that younger women when compared to their older counterparts were at greater risk for HPV infection and that this may be due to older women having acquired greater immunity to HPV infection [27,28].

Multiple infections were detected in 60.9% of HPV-positive women. It has been suggested that (i) women with multiple HPV genotypes may be more prone to persistent HPV infections, and (ii) there is interaction between genotypes resulting in the development of dysplasia and cancer [27,29]. Although in this group of women no specific patterns of co-infection were observed, women with cervical dysplasia had multiple infections consisting of high-risk and low-risk HPV genotypes. The high frequency of HPV infection, as well as the presence of multiple infections, is likely to contribute to the high incidence of invasive cervical cancer in Jamaica.

Although we found no relationship with HPV infection and other STIs (data not shown), a significant association was observed between the presence of bacterial vaginosis and multiple infections. Watts et al reported similar findings and suggested that changes in the vaginal flora may compromise cervical mucosa making it more vulnerable to HPV infections [30].

More pregnant, than non-pregnant women, in our population were found to acquire multiple infections. It is possible that pregnant women are at heightened susceptibility to infections as a result of the immunosuppression that occurs during pregnancy [31]. Pregnant women also, may be less likely to use condoms thus increasing their exposure to infections. In our study however, whereas pregnancy was associated with multiple infections in univariate analyses, with adjustment for bacterial vaginosis, pregnancy was no longer a predictor. Other studies have reported that prevalence of HPV infection among pregnant women increased with gestational age from 8.0% in the first trimester to 16.7% in the second and 23.1% in the third trimester [32,33] and that more than 2 vaginal deliveries were associated with a 3.9 fold increase in the development of cervical dysplasia [34]. Bazuaye et al. observed that in a population of Jamaican Colposcopy patients, parity was significantly associated with the development of cervical dysplasia [35].

## Conclusion

Our results indicate that genotype distribution in Jamaica differs from that found in Europe, North America and some parts of Asia. This has implications for the use of the recently developed vaccines, in Jamaica and similar populations. HPV genotyping as well as Pap smears should be considered for additional testing.

## Competing interests

The authors declare that they have no competing interests.

## Authors' contributions

NA conceived of the study, carried out statistical analyses and drafted the manuscript. AW was responsible for recruitment and data collection and laboratory assays. CR provided assistance and guidance with the HPV testing and genotyping. DG was responsible for recruitment and data collection at his practice. MJ and NY participated in design and statistical analyses. MS and HF advised on design and recruitment. All authors contributed to the writing of the manuscript and read and approved the final manuscript.
